# Oral health-related quality of life in an aging Canadian population

**DOI:** 10.1186/1477-7525-10-50

**Published:** 2012-05-15

**Authors:** Robert D Kotzer, Herenia P Lawrence, Joanne B Clovis, Debora C Matthews

**Affiliations:** 1 Discipline of Dental Public Health, Department of Biological and Diagnostic Sciences, Faculty of Dentistry, University of Toronto, 124 Edward Street, Toronto, Ontario M5G 1G6, Canada; 2 School of Dental Hygiene, Faculty of Dentistry, Dalhousie University, 5981 University Avenue, Halifax, Nova Scotia, B3H 4R2, Canada; 3 Department of Dental Clinical Sciences, Faculty of Dentistry, Dalhousie University, 5981 University Avenue, Halifax, Nova Scotia, B3H 1W2, Canada

**Keywords:** Oral health, Quality of life, Elderly, Aging, Seniors, Pre-seniors, Canada

## Abstract

**Background:**

The purpose of the study is to describe the impact of oral health-related quality of life (OHRQoL) on the lives of pre-seniors and seniors living in Nova Scotia, Canada.

**Methods:**

This cross-sectional study involved 1461 participants, grouped by age (pre-seniors [45–64] and seniors [65+]) and residential status (long-term care facility [LTC] or community). OHRQoL was measured using the 14-item Oral Health Impact Profile questionnaire (OHIP-14) in a random digit dialing telephone survey (for community residents) or a face-to-face interview (for LTC residents). Intra-oral examinations were performed by one of six dentists calibrated to W.H.O. standards.

**Results:**

Approximately one in four pre-seniors and seniors reported at least one OHRQoL impact ‘fairly/very often’. The most commonly reported impacts were within the dimensions ‘physical pain’ and ‘psychological discomfort’. It was found that 12.2% of LTC residents found it uncomfortable to eat any foods ‘fairly/very’ often compared to 7.7% in the community, and 11.6% of LTC residents reported being self-conscious ‘fairly/very often’ compared to 8.2% in the community. Of those residing in the community, pre-seniors (28.8%) reported significantly more impacts than seniors (22.0%); but there were no significant differences in OHRQoL between pre-seniors (21.2%) and seniors (25.3%) in LTC. Pre-seniors living in the community scored significantly higher than community dwelling seniors on prevalence, extent and severity of OHIP-14 scores. Logistic regression revealed that for the community dwelling sample, individuals living in rural areas in addition to those being born outside of Canada were approximately 2.0 times more likely to report an impact ‘fairly/very often’, whereas among the LTC sample, those having a high school education or less were 2.3 times more likely to report an impact.

**Conclusions:**

Findings indicate that the oral health and OHRQoL of both pre-seniors and seniors in LTC residents is poor. Community dwelling pre-seniors have the highest prevalence rate of oral impacts.

## Background

Compared to previous decades, the elderly population today is much more predominant in Canada and continues to rapidly increase due to longer life expectancy and the effects of the baby boom generation [1-3]. As these individuals (born between 1947 and 1966) begin to turn 65 years of age (in 2012), the number of seniors in Canada is estimated to jump from 4.2 million to 9.8 million from 2005 to 2036 [4]. In Nova Scotia, the seniors’ population in 2033 is estimated to be 257,874, an increase of 86.3% from 2007 [2]. Due to the aging of the population and increased purchasing power of today’s elderly, more people are taking advantage of the advancements in dental healthcare, leading to a decrease in rates of edentulism [5-8]. As a result of living longer and retaining more of their natural teeth, more oral problems arise and the treatment decisions of these patients becomes much more complex [5,9]. It is therefore imperative that information regarding the current oral health status, treatment needs, as well as the oral health-related quality of life (OHRQoL) of aging Canadians is collected in order to guide oral health policy. In the field of dentistry, the term “oral health-related quality of life” is commonly used to describe the impact that one’s oral health can have on their everyday life experiences [10,11]. The shift towards the importance of measuring one’s oral health-related quality of life reflects the reality that modern dentistry is not just aiming to prolong life or eliminate oral disease, but ultimately is attempting to make life better [12,13]. This study examines the differences in oral health-related quality of life between people aged 45 years and older living in the community and in long-term care (LTC) facilities in Nova Scotia. It also addresses the differences in oral health-related quality of life between pre-seniors and seniors within the community and LTC residences. Discussions regarding the disparities among these populations along with possible solutions to these problems are also explored.

## Methods

### Study Design

This cross-sectional study is part of a larger survey – The Oral Health of our Aging Population (TOHAP) – conducted in the province of Nova Scotia, Canada in 2008–09 [14]. The primary objective of TOHAP was to understand how the oral health and expectations of the baby boomer (45 yr-64 yr) generation differed from those preceding them (65 yr+) for the purpose of planning and creating policy. The participants were grouped by age (pre-seniors [aged 45 yr-64 yr] and seniors [65 yr+]), geographic location (urban or rural) and residential status (long-term care facility [LTC] or community dwelling). A pilot study was conducted prior to this survey to test the survey instruments and determine feasibility [15]. Ethics approval was received from the Health Sciences Research Ethics Board at Dalhousie University.

### Sample Size Determination

A sample size calculation based on known population prevalence rates determined the minimal sample size required was 382 pre-seniors, 382 seniors and 359 LTC residents [16]. This was adjusted to allow for a 10% cancellation rate of appointments.

### Sampling Frame and Sample Selection

All private and government owned LTC facilities with at least 20 beds per facility were included in the sampling frame. A total of 102 LTC facilities were used to determine the sampling frame. The LTC samples were proportionate to size (small, medium or large) as well as location (rural or urban). A small facility had 20–34 beds, a medium sized facility had 35–101 beds and a large facility had greater than 102 beds. The study was completed in 31 LTC facilities across 21 communities in Nova Scotia (NS), Canada. Four private facilities and 28 public facilities were sampled.

There are three kinds of publicly funded LTC facilities that are all licensed and approved by the NS Department of Health. Nursing Homes (homes for the aged) meet the needs of people who require a high level of personal care and professional nursing care. These facilities are licensed and inspected by the Department of Health. Residential Care Facilities are homes for people in need of supervision and limited help with personal care. These facilities are also licensed and inspected by the Department of Health. Community Based Options provide a similar level of care that residential care facilities offer but only accommodate a maximum of three people in each home. These facilities are unlicensed, but are inspected and approved by the Department of Health. No Community Based Options were included in this study because they did not meet the requirement of accommodating at least 20 beds. This study did not distinguish between Residential Care Facilities and Nursing Homes. LTC residents pay for accommodation charges (including salaries, benefits and operational costs of LTC employees) and personal expenses (including dental services and transportation) [17].

Community sites were selected based on proximity to previously selected LTC facilities. In total, 22 Nova Scotian communities were chosen.

### Measurements

Oral health-related quality of life was measured using the 14-item Oral Health Impact Profile questionnaire (OHIP-14) [18]. This questionnaire was administered through a random digit dialing telephone survey, completed by a Toronto-based telephone marketing company (for those living independently in the community), or a face-to-face interview (for those in LTC). The OHIP-14 was also translated into Acadian French (the local dialect). For each of the 14 items contained in the OHIP-14, study members were asked how often they had experienced the problem in the past year. Responses were coded as ‘very often’ (scoring 4), ‘fairly often’ (scoring 3), ‘occasionally’ (scoring 2), ‘hardly ever’ (scoring 1) or ‘never’ (scoring 0). This self-report questionnaire contains seven domains including: functional limitation, physical pain, psychological discomfort, physical disability, psychological disability, social disability and handicap.

In addition to the OHIP-14 questions, additional questions were derived from the 2007–09 Canadian Health Measures Survey [19]. These questions included: demographic information (age, sex, education, etc.), oral health questions (personal oral care habits and oral health care services utilization), general health questions, medication use, labour force activity (income and employment status) and questions regarding smoking and alcohol exposure.

Comprehensive intra-oral examinations were performed after the interview by one of six dentists, calibrated according to the standards of the World Health Organization (dentists were calibrated by a W.H.O. standardized dentist) [20] and the oral examination procedures used are reported elsewhere [14].

### Procedures

A call list was assembled for each community targeting pre-seniors and seniors living within a 20 km radius of the community. From each call list, numbers were chosen randomly and called until contact with the individual was made, or three calls were made without contact. Only those who were able to provide informed consent to complete the telephone survey and the clinical exam were included in the study. Informed consent was accepted in writing or verbally.

Once informed consent was obtained for community dwellers, an interview was done over the phone in either English or French, using a script developed for the study. However, none of the community residents and only 5 of the LTC residents completed the interview in French. There were no problems specific to the OHIP interview by telephone. The same telephone interviewers were used throughout the data collection period.

After the interview was conducted, participants scheduled appointments for a clinical exam within two weeks of the interview. Appointments for the clinical exams were scheduled using an online appointment system and conducted at hospitals, local private dental offices, long-term care facilities and public health offices. Approximately one-third of daily appointments were double booked to compensate for those who did not show up to their appointment. In LTC facilities interviews were conducted in person by a trained research assistant, followed immediately by an onsite clinical exam. As an incentive to complete the study, all participants were placed in contention to win one of two $250 prizes by means of a lottery (upon completion of the study). Data were collected in two different collection periods. The first collection period took place in the fall of 2008, and the second in the spring and summer of 2009. Further details related to the methodology of this study can be found in a separate publication [14].

### Data Analysis

Socio-demographic characteristics and self-perceived oral health status of community dwelling and LTC residents were summarized using descriptive statistics. Similarly, responses to individual OHIP-14 items were summarized according to place of residence. OHIP-14 overall scores were computed in three ways: (i) a total OHIP-14 score was calculated by summing responses over all 14 items, with possible scores ranging from 0 to 56 which indicates the severity of OHRQoL impacts; (ii) the prevalence of people reporting one or more items ‘fairly often’ or ‘very often’; (iii) the extent, which is the number of items reported ‘fairly often’ or ‘very often’ ranging from 0 to 14 [21]. Descriptive and inferential statistics were computed using SPSS version 19. The non-parametric Mann-Whiney *U* test was used to compare the mean extent and severity of oral health impacts between pre-seniors and seniors living in the community or in LTC residences. A chi-square analysis for categorical variables and logistic regression (using a stepwise and forward technique based on the Wald statistic) were used to identify factors related to prevalence of oral impacts for community dwelling and LTC residents. Statistical tests were two-tailed and interpreted at the 5% significance level. The variables that were inserted into the multivariate analysis and are thus being controlled for include: age, community type (rural vs. urban), sex, having a high school education, perceived general health, perceived quality of life, perceived mouth health, satisfaction with one’s teeth or dentures, frequency of dental visits, having dental insurance, smoking, household income, oral pain, dentate status and country of birth. The measures of severity and extent of OHRQoL impacts were not used as outcomes in multivariate analyses due to their skewed distributions.

## Results

Since there is such a small portion of Nova Scotia residents who live in LTC facilities (approximately 5%) [2], LTC residents were over-sampled in this study in order to gain enough power to identify an effect during statistical analyses. They represented 22.6% of the study population (Table 1). LTC residents were significantly more likely to be aged 65 and older, be female, edentulous, have a high school education or less, a household income of less than $30,000/yr, visit a dental professional less than once per year, brush their teeth less than twice per day (dentate only), floss their teeth less than once per day (dentate only) and were less likely to have dental insurance or be daily smokers (Table 1). In addition, LTC residents were significantly more likely to perceive their general health, quality of life and mouth health as fair or poor but have less oral pain than their community-dwelling counterparts (Table 2).

**Table 1:** Characteristics of study participants aged 45 years and older living in the community or long-term care in Nova Scotia, Canada

**Characteristic**		**% (N)**	
	**Total**	**Community**	**LTC**
	**100 (1461)**	**77.4 (1131)**	**22.6 (330)**
*Age (yrs)*			
45 – 64	45.3 (662)	**55.6 (629)**	**10 (33)**
65 and over	54.7 (799)	**44.4 (502)**	**90 (297)**
*Sex*			
Male	34.5 (504)	**37.1 (420)**	**25.5 (84)**
Female	65.5 (957)	**62.9 (711)**	**74.5 (246)**
*Education Level*			
More than high school	49.3 (718)	**57.6 (649)**	**21.0 (69)**
High school or less	50.7 (738)	**42.4 (478)**	**79.0 (260)**
*Community Type*			
Urban	59.1 (864)	59.2 (669)	59.1 (195)
Rural	40.9 (597)	40.8 (462)	40.9 (135)
*Annual Household Income*			
More than $30,000	57.6 (643)	**72.0 (618)**	**9.7 (25)**
Less than $30,000	42.4 (474)	**28.0 (240)**	**90.3 (234)**
*Dental Insurance*			
Yes	43.5 (621)	**50.7 (568)**	**17.3 (53)**
No	56.5 (806)	**49.3 (553)**	**82.7 (253)**
*Country of Birth*			
Canada	90.3 (1316)	90.3 (1018)	90.3 (298)
Other	9.7 (141)	9.7 (109)	9.7 (32)
*Frequency of Dental Visits*			
1+ times per year	59.9 (862)	**70.1 (782)**	**24.7 (80)**
< 1 times per year	40.1 (578)	**29.9 (334)**	**75.3 (244)**
*Dentate status*			
Dentate	81.8 (878)	**91.9 (684)**	**59.0 (194)**
Edentulous	18.2 (195)	**8.1 (60)**	**41.0 (135)**
*Brushing Frequency (dentate only)*			
2+ times per day	74.2 (650)	**79.3 (541)**	**56.2 (109)**
< 2 times per day	25.8 (226)	**20.7 (141)**	**43.8 (85)**
*Flossing Frequency (dentate only)*			
1+ times per day	35.2 (299)	**40.3 (268)**	**16.8 (31)**
< 1 times per day	64.8 (551)	**59.7 (397)**	**83.2 (154)**
*Smoking Frequency*			
*Occasionally or not at all*	88.4 (1288)	**87.3 (985)**	**92.1 (303)**
*Daily*	11.6 (169)	**12.7 (143)**	**7.9 (26)**

N.B. Bolded percentages are significant when p ≤0.05 using the Chi-square test.

**Table 2:** Self-perceived oral health status of adults aged 45 and older in Nova Scotia, Canada

**Characteristic**		**% (N)**	
	**Total**	**Community**	**LTC**
	**100 (1461)**	**77.4 (1131)**	**22.6 (330)**
*Perceived General Health*			
Excellent/ very good/ good	80.0 (1167)	**84.1 (950)**	**66.0 (217)**
Fair or poor	20.0 (292)	**15.9 (180)**	**34.0 (112)**
*Perceived Quality of Life*			
Excellent/ very good/ good	89.8 (1305)	**92.8 (1049)**	**79.0 (256)**
Fair or poor	10.2 (149)	**7.2 (81)**	**21.0 (68)**
*Perceived Mouth Health*			
Excellent/ very good/ good	79.9 (1161)	**81.1 (916)**	**75.6 (245)**
Fair or poor	20.1 (292)	**18.9 (213)**	**24.4 (79)**
*Oral Pain*			
No oral pain	69.0 (743)	**66.0 (493)**	**75.8 (250)**
Mouth, dental, jaw or other pain	31.0 (334)	**34.0 (254)**	**24.2 (80)**
*Satisfaction with Teeth/Dentures*			
Very satisfied/ satisfied/ neither	85.4 (1235)	85.4 (1235)	84.9 (269)
satisfied or dissatisfied	14.6 (211)	14.6 (211)	15.1 (48)
Dissatisfied or very dissatisfied			

N.B. Bolded percentages are significant when p ≤0.05 using the Chi-square test.

The most commonly reported oral health quality of life impacts were within the dimensions ‘physical pain’ and ‘psychological discomfort’ (Table 3). It was found that 12.2% of LTC residents found it uncomfortable to eat any foods ‘fairly often’ or ‘very often’ compared to 7.7% in the community. Nearly 12% of LTC reported being self-conscious ‘fairly/very often’ compared to 8.2% in the community, while 9.7% of LTC residents reported being embarrassed by their teeth, mouth or dentures ‘fairly/very often’ compared to 4% in the community. In addition, 6.1% of LTC residents compared to 2% of community dwellers reported impacts ‘fairly/very often’ with regards to difficulty pronouncing words.

**Table 3:** **Distribution of responses to individual OHIP-14 items and mean item scores (*n*** = 1460*)

**Dimension and description of item**	**Distribution of responses%**									**Mean (SD)**	
**“Because of trouble with your teeth, mouth or dentures during the last year, …”**	**Never (0)/Hardly Ever (1)**			**Occasionally (2)**			**Fairly Often (3)/Very Often (4)**				
	**Comm.**		**LTC**	**Comm.**	**LTC**		**Comm.**		**LTC**	**Community**	**LTC**
*Functional limitation*											
have you had trouble pronouncing any words?	92.9		86.7	5.2	7.3		2.0		6.1	0.09 (0.35)	0.19 (0.53)
have you felt that your sense of taste has worsened?	88.5		88.8	7.5	5.9		4.0		5.3	0.16 (0.46)	0.17 (0.49)
*Physical pain*											
have you had painful aching in your mouth?	79.7		83.6	15.2	11.8		5.2		4.5	0.25 (0.54)	0.21 (0.51)
have you found it uncomfortable to eat any foods?	74.8		73.3	17.5	14.6		7.7		12.2	0.33 (0.61)	0.39 (0.69)
*Psychological discomfort*											
have you been self-conscious?	78.7		74.5	13.1	14.0		8.2		11.6	0.29 (0.61)	0.37 (0.68)
have you felt tense?	80.2		84.4	12.4	8.0		7.4		7.6	0.27 (0.59)	0.23 (0.58)
*Physical disability*											
has your diet been unsatisfactory?	86.3		84.5	7.8	7.9		5.9		7.6	0.20 (0.53)	0.23 (0.58)
have you had to interrupt meals?	91.0		88.8	6.0	6.7		2.9		4.6	0.12 (0.41)	0.16 (0.47)
*Psychological disability*											
have you found it difficult to relax?	85.4		88.4	9.6	6.4		5.1		5.2	0.20 (0.51)	0.17 (0.49)
have you been a bit embarrassed?	83.8		82.4	12.2	7.9		4.0		9.7	0.20 (0.49)	0.27 (0.63)
*Social disability*											
have you been a bit irritable with other people?	86.1		89.1	10.6	7.3		3.3		3.6	0.17 (0.46)	0.15 (0.45)
have you had difficulty doing your usual jobs?	93.8		93.9	3.9	3.1		2.3		3.1	0.09 (0.35)	0.09 (0.38)
*Handicap*											
have you felt that life in general was less satisfying?	89.7		87.2	6.6	6.7		3.7		6.1	0.14 (0.44)	0.19 (0.53)
have you been totally unable to function?	96.1		95.1	2.6	2.4		1.3		2.4	0.05 (0.28)	0.07 (0.34)

*One participant in the community sample did not have complete data for the OHIP-14.

In terms of ‘prevalence’ of impact, 25.8% of the community dwellers and 24.8% of LTC residents reported one or more OHIP problems ‘fairly/very often’. A larger percentage of LTC residents reported one or more impacts ‘fairly/very often’ in the functional limitation, physical pain, psychological disability and handicap dimensions. Regarding ‘extent’ of impact, (*i.e.*, the mean number of OHIP items reported ‘fairly/very often’) the mean for community residents was 0.63 (SD = ±1.59) and 0.89 (SD = ±2.24) for LTC residents. Furthermore, in terms of ‘severity’ of impact, (*i.e.*, the total OHIP score) the mean was 5.57 (SD = ±7.57) for community dwellers and 5.57 (SD = ±9.58) for LTC residents (Table 4). However, a statistically significant difference was reported in terms of the mean number of items reported ‘fairly/very often’ between community and LTC residents.

**Table 4:** **Prevalence, extent and severity of impacts by OHIP-14 subscale and total score (*n*** **= 1460)**

**Dimension**	**Prevalence: % reporting 1+ impacts fairly/very often (no.)**		**Extent: mean no. of items reported fairly/very often (SD)**		**Severity: mean OHIP-14 score (SD)**	
	**Community**	**LTC**	**Community**	**LTC**	**Community**	**LTC**
Functional limitation	**5.4 (61)**	**9.4 (31)**	**0.06 (0.26)**	**0.11 (0.37)**	**0.56 (1.20)**	**0.69 (1.48)**
Physical pain	**9.6 (109)**	**13.9 (46)**	0.13 (0.42)	0.17 (0.44)	1.25 (1.81)	1.17 (1.85)
Psychological discomfort	12.0 (136)	13.6 (45)	**0.15 (0.45)**	**0.19 (0.51)**	1.16 (1.86)	1.14 (2.03)
Physical disability	7.6 (86)	7.9 (26)	**0.09 (0.33)**	**0.12 (0.44)**	**0.69 (1.40)**	**0.76 (1.78)**
Psychological disability	**7.3 (82)**	**11.5 (38)**	**0.09 (0.34)**	**0.15 (0.44)**	0.85 (1.59)	0.85 (1.81)
Social disability	4.7 (53)	4.2 (14)	0.06 (0.27)	0.07 (0.33)	**0.59 (1.32)**	**0.46 (1.32)**
Handicap	**4.1 (46)**	**6.7 (22)**	**0.05 (0.26)**	**0.09 (0.34)**	**0.47 (1.19)**	**0.51 (1.43)**
Total OHIP-14 score	25.8 (291)	24.8 (82)	**0.63 (1.59)**	**0.89 (2.24)**	5.57 (7.57)	5.57 (9.58)

N.B. Bolded percentages are significant when p ≤0.05 using the Chi-square test.

N.B. Bolded means are significant when p ≤0.05 using the Independent Samples T-test.

Further analysis of prevalence, extent and severity were carried out by comparing pre-seniors with seniors in both LTC and community settings (Table 5). It was found that in the community, pre-seniors scored significantly higher than seniors on prevalence (*p* = 0.009), extent (*p* = 0.007) and severity (*p* < 0.001). In the LTC residences, seniors scored higher than pre-seniors for prevalence, extent and severity but there was not a statistically significant difference in the OHIP-14 scores. Although not mentioned in the tables, pre-seniors from the community were compared with pre-seniors in LTC, and seniors from the community were compared with seniors from LTC. Pre-seniors in the community scored higher on prevalence, extent and severity than pre-seniors in LTC residences, but severity was the only significant difference (*p* = 0.033). Furthermore, seniors in LTC residences scored higher than seniors in the community for prevalence, extent and severity, but the differences were not statistically significant.

**Table 5:** Prevalence, extent and severity of impacts by OHIP-14 subscale grouped by pre-seniors and seniors

	**Community**			**LTC**		
	**Pre-seniors** **(n=629)**	**Seniors** **(n=501)**	**P-value**	**Pre-seniors** **(n=33)**	**Seniors** **(n=297)**	**P-value**
*Prevalence*: % reporting 1+ impacts fairly/very often (no.)	28.8 (181)	22.0 (110)	0.009^*^	21.2 (7)	25.3 (75)	0.610^*^
*Extent*: mean no. of items reported fairly/very often (SD)	0.73 (1.73)	0.49 (1.40)	0.007^**^	0.45 (1.33)	0.94 (2.32)	0.456^**^
*Severity*: mean OHIP-14 score (SD)	6.22 (8.0)	4.75 (6.92)	<0.001^**^	4.30 (7.29)	5.71 (9.80)	0.867^**^

^*^P-value obtained from the Chi-squared test.

^**^P-value obtained from the Mann–Whitney *U* test.

Bivariate analyses were conducted for prevalence of impacts for both community and LTC residents. Community residents who reported one or more impacts ‘fairly often’ or ‘very often’ were more likely to be pre-seniors, live in a rural area, be female, have a high school education or less, make less than $30,000 per year, visit the dentist less than once per year, smoke daily, have oral pain, perceive their general health, mouth health and quality of life to be fair or poor and be dissatisfied with their teeth or dentures (Table 6). LTC residents who reported one or more impacts ‘fairly often’ or ‘very often’ were more likely to have a high school education or less, have oral pain, perceive their general health, mouth health and quality of life to be fair or poor and be dissatisfied with their teeth or dentures (Table 7).

**Table 6:** Bivariate analyses for prevalence of impacts (‘fairly often’ or ‘very often’) for community residents

**Characteristic**	**% (N)**		
	**No Impacts**	**1+ Impacts**	**P-value**
*Age (yrs)*			
45 – 64	71.2 (448)	28.8 (181)	0.009
65 and over	78.0 (391)	22.0 (110)	
*Community Type*			
Urban	79.2 (529)	20.8 (139)	< 0.001
Rural	67.1 (310)	32.9 (152)	
*Sex*			
Male	78.3 (328)	21.7 (91)	0.017
Female	71.9 (511)	28.1 (200)	
*Education Level*			
More than high school	78.3 (508)	21.7 (141)	< 0.001
High school or less	68.6 (328)	31.4 (150)	
*Annual Household Income*			
More than $30,000	77.0 (476)	23.0 (142)	< 0.001
Less than $30,000	64.2 (154)	35.8 (86)	
*Dental Insurance*			
Yes	75.9 (431)	24.1 (137)	0.278
No	73.1 (404)	26.9 (149)	
*Country of Birth*			
Canada	74.8 (761)	25.2 (256)	0.341
Other	70.6 (77)	29.4 (32)	
*Frequency of Dental Visits*			
1+ times per year	77.7 (608)	22.3 (174)	< 0.001
< 1 time per year	65.6 (219)	34.4 (115)	
*Dentate Status*			
Dentate	76.3 (524)	23.7 (163)	0.169
Edentulous	68.3 (41)	31.7 (19)	
*Smoking Frequency*			
Occasionally or not at all	75.8 (747)	24.2 (238)	0.003
Daily	64.3 (92)	35.7 (51)	
*Oral Pain*			
No	82.8 (408)	17.2 (85)	< 0.001
Yes	61.8 (157)	38.2 (97)	
*Perceived General Health*			
Excellent/ very good/ good	77.9 (740)	22.1 (210)	< 0.001
Fair or poor	55.0 (99)	45.0 (81)	
*Perceived Mouth Health*			
Excellent/ very good/ good	79.7 (730)	20.3 (186)	< 0.001
Fair or poor	50.7 (108)	49.3 (105)	
*Satisfaction with Teeth or Dentures*			
*Satisfied*	79.5 (768)	20.5 (198)	< 0.001
*Dissatisfied*	42.9 (70)	57.1 (93)	
*Perceived Quality of Life*			
*Excellent/ very good/ good*	76.5 (803)	23.5 (246)	< 0.001
*Fair or poor*	44.4 (36)	55.6 (45)	

**Table 7:** Bivariate analyses for prevalence of impacts (‘fairly often’ or ‘very often’) for LTC residents

	**% (N)**		
**Characteristic**	**No Impacts**	**1+ Impacts**	**P-value**
45 – 64	78.8 (26)	21.2 (7)	0.610
65 and over	74.7 (222)	25.3 (75)	
Urban	76.9 (150)	23.1 (45)	0.371
Rural	72.6 (98)	27.4 (37)	
*Sex*			
Male	73.8 (62)	26.2 (22)	0.742
Female	75.6 (186)	24.4 (60)	
*Education Level*			
More than high school	84.1 (58)	15.9 (11)	0.052
High school or less	72.7 (189)	27.3 (71)	
*Annual Household Income*			
More than $30,000	88.0 (22)	12.0 (3)	0.081
Less than $30,000	71.8 (168)	28.2 (66)	
*Dental Insurance*			
Yes	79.2 (42)	20.8 (11)	0.384
No	73.5 (186)	26.5 (67)	
*Country of Birth*			
Canada	74.8 (223)	25.2 (75)	0.682
Other	78.1 (25)	21.9 (7)	
*Frequency of Dental Visits*			
1+ times per year	76.3 (61)	23.8 (19)	0.766
< 1 time per year	74.6 (182)	25.4 (62)	
*Dentate Status*			
Dentate	75.3 (146)	24.7 (48)	0.957
Edentulous	75.0 (102)	25.0 (34)	
*Smoking Frequency*			
Occasionally or not at all	76.2 (231)	23.8 (72)	0.096
Daily	61.5 (16)	38.5 (10)	
*Oral Pain*			
No	79.6 (199)	20.4 (51)	0.001
Yes	61.3 (49)	38.8 (31)	
*Perceived General Health*			
Excellent/ very good/ good	81.1 (176)	18.9 (41)	0.001
Fair or poor	64.3 (72)	35.7 (40)	
*Perceived Mouth Health*			
Excellent/ very good/ good	85.7 (210)	14.3 (35)	< 0.001
Fair or poor	40.5 (32)	59.5 (47)	
*Satisfaction with Teeth or Dentures*			
*Satisfied*	81.0 (218)	19.0 (51)	< 0.001
*Dissatisfied*	56.3 (27)	43.8 (21)	
*Perceived Quality of Life*			
*Excellent/ very good/ good*	80.5 (206)	19.5 (50)	< 0.001
*Fair or poor*	54.4 (37)	45.6 (31)	

Logistic regression models controlling for all the factors (significant and non-significant) at the bivariate level of analysis were used to predict the prevalence of impacts ‘fairly often’ or ‘very often’ for community and LTC residents, separately. For the community dwelling sample, individuals living in rural areas and those born outside of Canada were approximately 2.0 times more likely to report an impact ‘fairly/very often’ (Table 8). Having oral pain, fair or poor perceived mouth health and dissatisfaction with teeth or dentures also caused community residents to report impacts. Among the LTC sample, those having a high school education or less were 2.3 times more likely to report an impact ‘fairly often’ or ‘very often’ (Table 9). Those with fair or poor perceived mouth health were nearly 10 times more likely to report impacts ‘fairly often’ or ‘very often’.

**Table 8:** Logistic regression model for prevalence of impacts (‘fairly often’ or ‘very often’) for community residents

	**Adjusted Odds Ratio**	**95% CI for Odds Ratio**	**P-value**
Living in a rural area	2.07	1.35 – 3.17	0.001
Having oral pain	1.87	1.21 – 2.88	0.005
Born outside of Canada	1.97	1.01 – 3.85	0.048
Fair or poor perceived mouth health	2.19	1.30 – 3.71	0.003
Dissatisfaction with teeth or dentures	5.16	2.87 – 9.27	<0.001

CI = Confidence Interval.

**Table 9:** Logistic regression model for prevalence of impacts (‘fairly often’ or ‘very often’) for LTC residents

	**Adjusted Odds Ratio**	**95% CI for Odds Ratio**	**P-value**
High school education or less	2.29	1.05 – 5.00	0.039
Fair of poor perceived mouth health	9.49	5.27 – 17.09	<0.001

CI = Confidence Interval.

## Discussion

The 2007–09 Canadian Health Measures Survey, in accordance with Statistics Canada, released data regarding the oral health status and treatment needs of elderly Canadians, but did not do so at the provincial level [22]. The TOHAP study is the first to focus on the oral health of older adults living in the province of Nova Scotia. The findings of this study are not only important in assembling a complete picture of the oral health of Canadians, but they also provide important insight into the oral health-related quality of life of these individuals.

The most interesting finding of this study was regarding the comparison of oral health impacts on pre-seniors and seniors. It was found that pre-seniors living in the community reported more oral health impacts than seniors even though the oral health of pre-seniors was better than that of seniors [23]. This reinforces the notion that individual expectations and experiences can greatly impact ones satisfaction or dissatisfaction with their oral health [12]. For example, one who experiences poor health but has low expectations may not perceive their health to have a significant impact on his/her life. Seniors living within the community may not feel as though oral health has a huge impact on their lives and may be more satisfied with the quality of their oral health compared to their general health, causing them to report less impacts ‘fairly often’ or ‘very often’. In contrast, one who has excellent oral health but extremely high expectations might report being dissatisfied due to a minor oral health-related problem. Community dwelling pre-seniors who are generally in good health may become irritated by small oral health problems, and frustrated that dental visits can be expensive and cut into work hours [12]. Locker and Gibson’s (2005) study of community living individuals over the age of 50 reported that 16.5% of those who rated their oral health as either excellent, very good, or good were dissatisfied with their oral health [12]. Moreover, 50.8% of participants who rated their oral health as fair or poor reported that they were satisfied with mouth, teeth or gums.

In addition, the frames of reference on which people base their oral health can naturally range depending on a host of variables. While some compare themselves to others who are close in age, others might use their physical or emotional state to assess their oral health. Some people who have, or perceive themselves as having, poor oral health may actually be satisfied with the state of their oral health [12,24]. Sprangers and Schwartz (1999) explain this phenomenon through the process of response shift. This is when changes in internal standards, values and meanings of health contribute to the acceptance of an individual’s illness or disability [24]. As individuals age, they are more likely to consider minor or even severe oral health problems as insignificant at this point in their lives [12]. The theory of response shift may explain why community dwelling seniors, and the elderly population in general, may report fewer impacts in certain dimensions [24]. As these individuals age, they come to accept that their health is deteriorating and they may consider oral health problems as less significant [12]. Consequently, these oral health problems take a backseat to general health problems. A study completed in Ontario involving 61 residents in three long-term care facilities suggests that general health issues often overshadowed and minimized oral health issues in long-term care facilities. Chronic illnesses such as Alzheimer’s and Parkinson’s disease, which interfere with ones cognitive and communicative skills, cause barriers in identifying treatments needs for these residents [25].

In the study completed by Locker and Quinonez [26] telephone numbers for households (therefore those living in the community) were randomly sampled in a Canadian population. They found that those between the ages of 35–54 reported an 18.3% prevalence rate of oral impacts, and those aged greater than or equal to 55 years reported a 19.5% prevalence rate [26]. In this national study an older population of community dwellers reported 1.2% more impacts than a younger population of community dwellers. Despite the slight difference in age groups, our results show that among those living within the community, pre-seniors reported a 28.8% prevalence rate of oral impacts, whereas seniors reported a prevalence rate of 22.0%.

Another important finding of our study indicates that approximately one in four pre-seniors and seniors report at least one or more impacts of their oral health on the quality of life ‘fairly’ or ‘very often’. This is slightly higher than a national study of adults aged 55 years and older where the finding was one in five (19.5%) [26]. The Yukon, Nunavut and Northwest Territories, which make up 0.3% of the Canadian population, were not included in that study. It is evident that no matter where you live in Canada, people in your community are going to report having oral impacts. But, it is important to note that older samples and edentulous samples will report having more OHRQoL impacts.

Logistic regression models indicate that both socio-demographic factors and self-perceived oral health can have an effect on the prevalence of impacts. The findings that pre-seniors and seniors in rural areas have the poorest OHRQoL suggest that a decreased access to dental care may be affecting their oral health and OHRQoL. Further findings show that elderly residents living in the community visit the dentist significantly less often if they live in rural areas as opposed to urban areas. Results indicate that 75.4% of Nova Scotia residents aged 45 and older who live in an urban area visit the dentist one or more times per year, whereas only 62.4% of rural residents visit the dentist one or more times per year. According to the literature, “in dentistry, a functional definition of an elderly adult is based on his or her ability to travel to seek services” [5]. Many elderly patients who live in rural areas may have access to fewer dental clinics, or there may be barriers limiting their access to care. Barriers include lack of public transportation, cost of transportation and treatment, or mobility issues [27]. The reliance of many seniors on others for help may also limit their ability to receive dental care [27].

Furthermore, funding for retired employees must be developed by union negotiators, working Canadians must plan for retirement by saving money for dental care and family members and caregivers must be educated in the importance of dental care for the elderly [28].

Although many health economists believe that government funding may be insufficient to meet the increasing dental needs of the baby boom population [28], education is a relatively inexpensive, yet effective dental health care initiative because it is generally less expensive to prevent disease than cure it [28]. A large percentage of the Nova Scotian elderly population has a high school education or less. This is especially troubling because education is a social determinant of health and education is also highly related to health literacy.

Health literacy has been defined as “the degree to which individuals have the capacity to obtain, process, and understand basic health information and services needed to make appropriate health decisions” [29]. Health literacy skills are essential in the maintenance of quality of life for the elderly population [30]. It has also been shown to be an important contributor to both general and oral health [28]. As individuals age, health literacy also becomes an important tool to help take or administer medications appropriately [31].

Although our study, in addition to other similar studies, have identified potential correlates of health literacy, few studies have attempted to recognize educational and learning pathways that increase health literacy skills throughout ones life [30]. The development and maintenance of health literacy skills throughout ones life can be accomplished by the use of adult education, seminars, self-study, internet use, library use, daily reading and engagement in social networks [30]. A study found that practicing literacy at home by methods such as reading books, magazines and newspapers, had a stronger effect on ones health literacy than educational attainment [32]. These practices can be maintained throughout ones life and will maintain and increase ones literacy in a relatively inexpensive manner. In addition, using the internet or computer to learn was found to be one of the strongest predictors of adequate health literacy [30].

Another issue that must be addressed is the current level of communication between dental care providers and their patients, which is important for the elderly population. Effective communication is critical for dentists and hygienists in improving the oral health literacy of their patients [33]. Several findings suggest that the communication techniques used by dentists may not be effectively accommodating the literacy skills of certain patients [34]. One technique among others that has been proven to be effective in increasing the health literacy of patients is the teach-back method; therefore it is important that a set of communications guidelines for practicing dentists be developed [34].

While some studies question whether literacy is really a problem in the context of health care, and suggest a need for more Canadian research in this area [35], education and health literacy can improve access to care for Canadian seniors and the general population by focusing on health, oral health, and quality of life issues [28]. Education that focuses and raises awareness on how oral health enhances self-image and social interactions can also positively affect attitudes towards care [28].

In addition, it was found that those who were born outside of Canada living within the community have greater oral health impacts, implying that oral health literacy, understanding the Canadian health-care system and acculturation may be limiting their access to dental care. An increase in educational resources and training by dentists and dental hygienists can be essential in developing proper oral health care skills and routines for seniors, LTC nursing staff, and family members [27]. Education is also necessary so that they can provide care in a productive, cost-effective and timely manner [28].

The binary logistic regression model also indicated that for those living in the community, people with oral pain were 1.87 times more likely to report impacts, and those with fair or poor perceived mouth health were 2.19 times more likely to report impacts. These two variables are closely related to the outcome of oral health-related quality of life and it is therefore not surprising to find them in this model. Similarly, those with dissatisfaction with the appearance of their teeth and/or dentures were 5.16 times more likely to report impacts ‘fairly often’ or ‘very often’. This readdresses the theme of how a complication with ones teeth and/or dentures can have a significant impact on oral health-related quality of life. Dissatisfaction with the appearance of teeth and/or dentures is directly related to variables on the OHIP-14 such as being self conscious and embarrassed. Being self conscious because of trouble with teeth, mouth or dentures was one of the highest scoring items on the OHIP-14.

Moreover, LTC residents with a low education level may be a group at risk in terms of greater impacts on their OHRQoL. LTC residents have poorer indicators of socioeconomic status including household income and dental insurance. Since dental coverage is not covered by the Canadian healthcare system, out of pocket costs may deter people from seeking dental care or accepting recommended dental care when visiting the dentist [22]. The Canadian Health Measures Survey reported that as Canadians age they are less likely to have dental insurance. In addition, being born outside of Canada, annual income and level of education are also directly related to having dental insurance [22]. A 2006 study using Canadian health survey data from 2003, found that the probability of receiving any dental care throughout the course of a year increases dramatically with dental insurance, household income and level of education [36]. This study confirms these findings in the NS population as 79% of LTC residents have less than or equal to a high school education, 82.7% do not have dental insurance and 90.3% have an annual household income of less than $30,000.

Reported in the 2006 census, only 24% of adults aged 25–64 had a high school diploma as their highest level of educational attainment, while 15% did not graduate from high school. In addition, 32% of adults aged 55 to 64 years did not have a high school diploma [37]. Educational attainment is recognized as one of the key components of socioeconomic status, and while income and education are highly correlated, education is an independent predictor of health status and visiting the dentist [38,39]. Regardless of age, people with low education levels have more disabilities and chronic illnesses [38]. People with a higher educational background tend to embrace positive health practices and have access to healthier physical environments [38].

It is clear that public health initiatives need to focus on Canadians with low levels of education. Even though access to education and literacy levels are for the most part managed outside of the health sector, they have a direct effect on health status. Therefore, multi-sectoral strategies must be implemented in order to improve the health of Canadians [38].

In addition to a high school education, LTC residents with fair or poor perceived mouth health were 9.49 times more likely to report impacts. LTC residents have poor oral hygiene and limited access to routine dental care [40]. It has been shown that once a comprehensive dental program is implemented into LTC facilities, residents who receive dental care show improvements in caries rates, periodontal health, and other clinical oral disorders [40]. Living in an LTC facility is a barrier to treatment in and of itself. Therefore it is imperative that dental programs be developed in order to increase access to dental care for seniors in LTC, by providing transportation or by bringing oral care providers and dental equipment into the facilities.

## Conclusions

This study has provided valuable information regarding the oral health-related quality of life of pre-seniors and seniors living in Nova Scotia, Canada. LTC residents are more likely to have poorer indicators of socio-demographic characteristics and self-perceived oral health status compared to community dwellers. Having more oral health problems can have an effect on one’s OHRQoL and in turn, explain why LTC residents report impacts ‘fairly/very often’ on the OHIP-14. One in four pre-seniors and seniors living in the community and LTC facilities reported one or more impacts ‘fairly/very often’, however, pre-seniors in the community experienced greater prevalence, extent and severity of oral impacts than seniors. This finding suggests that as people age, oral health problems may take a backseat to general health problems. The study findings also indicate that pre-seniors and seniors in rural areas have the poorest OHRQoL, suggesting that a decreased access to dental care may be affecting their oral health and OHRQoL. In addition, lower levels of education and health literacy overall and especially among those who were born outside of Canada but are now living within the community have greater oral health impacts, suggesting that decreased oral health literacy and a lack of understanding of the Canadian health care system may be limiting their access to dental care. LTC residents with a low education level and low health literacy may be a group at higher risk in terms of greater impacts on their OHRQoL.

## Abreviations

OHRQoL: Oral health-related quality of life; TOHAP: The Oral Health of our Aging Population; LTC: Long-term care; OHIP: Oral Health Impact Profile; NS: Nova Scotia.

## Competing interests

The authors declare that they have no competing interests.

## Authors' contributions

RK carried out the data analysis and the interpretation of data as well as wrote the manuscript. HPL contributed to the study design, calculated the required sample size and random selection of the long-term care facilities. She also participated in the analysis and interpretation of the data and critically reviewed the manuscript. DCM and JBC were principal investigators of the TOHAP study. They contributed to the interpretation of the data and review of the manuscript. All authors read and approved the final manuscript.
